# Strengthening RC beams and columns with CFRP, GFRP and KFRP laminates

**DOI:** 10.1038/s41598-026-43464-1

**Published:** 2026-03-31

**Authors:** Karim Adel, Moaz Abdelazeem, Ahmed Sherif, Mohanad Saadeldin, Hossam Rashed, Mohamed M. Omran, Nouran M. Tawfik

**Affiliations:** 1 Civil & Construction Engineering, School of Engineering & Applied Science, Nile University, Giza, Egypt; 2 Smart Engineering Systems Center, SESC, Nile University, Giza, Egypt

**Keywords:** Reinforced concrete strengthening, Fiber reinforced polymers, Natural fibers, Finite element modeling, Plastic damage model, Hashin damage, Engineering, Materials science

## Abstract

Prolonged exposure to adverse conditions affects the performance and promotes the degradation of reinforced concrete (RC) structures, requiring repair and strengthening to preserve their integrity and functionality. Synthetic fibers, including carbon fiber reinforced polymer (CFRP) and glass fiber reinforced polymer (GFRP), are frequently employed for retrofitting owing to their superior strength-to-weight ratio and simple installation. Natural fibers, such as kenaf, have been increasingly incorporated into fiber reinforced polymer (FRP) composites as sustainable alternatives in the construction industry due to their lightweight nature and low carbon footprint. Yet, many investigations focused on synthetic fibers, thus, the purpose of this study is to develop finite element (FE) models for RC beams and columns retrofitted with CFRP, GFRP and kenaf fiber reinforced polymer (KFRP), to examine the impact of natural fibers and comparing the findings with those derived from synthetic fibers. The models consider element types, mesh discretization, solution methodologies, and nonlinearities. Concrete behavior is represented using Concrete Damage Plasticity (CDP), whereas fiber laminates employ the Hashin damage model. The FE model’s load-displacement behavior, ultimate strength, and failure mechanisms were verified against existing experimental and numerical findings. The findings indicate that FRP wrapping substantially enhances the load-carrying capacity of RC beams, with ultimate load increases ranging from approximately 13% for KFRP to 66% for CFRP, whereas the corresponding improvements for RC columns are notably smaller, remaining below 7%. Although KFRP exhibits lower mechanical performance than GFRP and CFRP, its sustainability and cost-effectiveness support its use in applications where environmental and economic considerations are prioritized.

## Introduction

Extended service of reinforced concrete (RC) structures in complex environments affects performance and degradation^1–3^. Exposure to harsh environmental conditions, excessive loading from structural upgrades, design or construction deficiencies, reinforcing steel bar corrosion, earthquake damage, and fire can cause structural members to deteriorate and lose stiffness or strength over time. Therefore, several RC structures need repair and strengthening, which have been shown to effectively address deterioration. Fiber reinforced polymer (FRP) are one of the most successful methods for reinforcing deteriorated sections and restoring structural integrity. Its exceptional strength-to-weight ratio, easy installation, corrosion resistance, adaptability, decreased life-cycle costs, and longevity make this strengthening system efficient. Due to this preference, it strengthens better than steel plates, section expansion, and concrete jacketing. Most RC constructions are strengthened with carbon fiber reinforced polymer (CFRP) and glass fiber reinforced polymer (GFRP). Carbon fiber is preferred for applications requiring strength, rigidity, and longevity, especially in harsh circumstances. Glass fiber is cheaper and better for modest strengthening or low-budget tasks. The choice depends on the RC structure’s load requirements, environmental considerations, and project budget. The strengthening system uses externally bonded materials on the structural element to adapt the structure for new functions, according to ACI 440-2R^4^, CNR DT-200 R1^5^, and Fib-Bulletin 14^6^. FRP plates or sheets can be easily bonded to RC elements using wet lay-up with epoxy resin/adhesive. Shear, flexural, and axial strengthening of RC beams and columns are achievable with FRP sheets or plates. Despite being a strategy extensively examined in numerous studies, certain concerns require additional investigation, as evidenced by many recent publications. Khan & Fareed^7^. examined the flexural performance of RC beams strengthened by externally bonded CFRP wraps, varying the shear span to depth ratio. The study revealed that RC beams with CFRP at the bottom and sides, with or without end anchorages, improved stiffness, ductility, and load capacities. The shear strengthening of RC beams was studied using U-Wrapped and fully wrapped CFRP laminates by Mhanna et al^8^. The testing findings showed that complete-wrapping improves shear strength and ductility of RC beams more than U-wrapping of equal depth. Shear strengthening on RC T-beams using CFRP U-wraps with spike anchors was studied by Abdallah^9^. RC T-beams strengthened with CFRP U-wraps have 45% higher shear capability. Anchoring the U-jackets increased FRP shearing capability by 27–55% over unanchored U-wraps. de Freitas Arcine. et al.^10^ used experimental and numerical approaches to study how internal stirrups and outside CFRP strengthening affect shear in RC beams. All beams with laminates above stirrups showed shear failure. However, beams joined between stirrups failed due to bending and shear loads. Al Shboul et al.^11^ used FRP layers in tension and compression zones with U-wrap anchors to improve load-carrying capacity, stiffness, ductility, and serviceability. Assad et al.^12^ tested strengthened and anchored RC beams’ flexural behavior. CFRP laminates and splay anchors increased specimen flexural strength. Compared to a control unstrengthen specimen and an unanchored strengthened specimen, load-carrying capacity increased by 60–104% and 14–28%, respectively. Wang et al.^13^ performed four-point bending tests on four beams, unstrengthen and strengthened with various CFRP configurations, to evaluate and assess the flexural load-carrying improvements of the strengthened beams. They also created numerical models using a unified modelling strategy and evaluated them against experimental results. Zhang et al.^14^ used three-dimensional meso-scale numerical modelling to study the shear failure of stirrup-free CFRP RC beams. According to the study, the CFRP fiber ratio affects RC beam shear strength. Akkaya et al.^15^ tested twenty-two CFRP and GFRP-wrapped deep beams. The beams were tested with different FRP layers and spacings. The FRP strips improved beam shear and ductility.

Khan and Fareed^16^ conducted an experimental study to investigate the performance of CFRP-wrapped RC columns under uniaxial compression. They concluded that the RC specimens wrapped in CFRP demonstrated superior compressive strength relative to other CFRP configurations, achieving strength enhancements of 65% and 20% compared to plain concrete and RC columns, respectively. Lotfy and Ahmed^17^ conducted an experimental and numerical investigation on the strengthening of short RC columns with various cross sections using GFRP jackets. Both analyses indicate that externally applied FRP sheets are crucial for enhancing the strength and ductility of short concrete columns. Furthermore, they introduced a design equation to determine the ultimate axial load for short columns strengthened by a complete GFRP wrap. Ganesh et al.^18^ executed an experimental program to examine the performance of RC columns wrapped in CFRP. Under eccentric and concentric loading, it was established that CFRP wrap possesses significant load-bearing capacity in RC components, even under eccentric loading conditions. Li et al.^19^ developed a finite element (FE) model to simulate CFRP-retrofitted RC columns subjected to blast loading, incorporating the strain rate effects on both the concrete and steel reinforcement, as well as the FRP composite. Moghtadernejad et al.^20^ proposed the use of external confinement by FRP to address inadequacies in concrete columns resulting from fire damage. Rectangular columns with heat damage are wrapped in one or two layers of CFRP and GFRP. The experimental results demonstrate that the ultimate axial load capacity of post-heated columns, reinforced with two layers of FRP, significantly approached the axial load capacity of unheated columns. More recently, advanced confinement strategies have been investigated to further elucidate the axial behavior of FRP-confined RC columns. In particular, Wang et al.^21^ conducted an experimental and numerical study on RC columns confined using CFRP strip ties, examining the influence of confinement configuration on axial compressive strength and ductility. Their findings demonstrated that the effectiveness of FRP confinement is strongly dependent on the confinement layout and stress transfer mechanism, with non-uniform confinement leading to limited strength enhancement compared to fully effective confinement schemes. These observations further confirm that, while FRP wrapping can improve the axial performance of RC columns, the degree of improvement is highly sensitive to confinement efficiency and structural configuration.

Synthetic fibers such as carbon and glass fibers are frequently employed to retrofit RC constructions. Natural fibers can serve as a more sustainable alternative to synthetic fibers and have lately been integrated into the building sector due to their benefits, including cost-effectiveness, lightweight properties, corrosion resistance, and minimal carbon footprint. The heightened worldwide consciousness regarding environmental preservation and new legislation have driven researchers to create more eco-friendly FRP materials as substitutes for synthetic FRP materials. Researchers are currently doing experimental and numerical examinations to examine the behavior of RC elements retrofitted with natural fibers. Nwankwo and Ede^22^. performed experimental and numerical investigations to examine the strengthening of concrete beams using natural FRP laminates, specifically kenaf fiber reinforced polymer (KFRP) laminate, which was designed and produced to enhance the flexural capacity of a RC beam as a more sustainable substitute for synthetic fibers. The computational analysis revealed that the KFRP laminate enhanced the beams’ load-carrying capability by 77.9%. The KFRP laminate similarly diminished beam deflections at equivalent loads. Makhlouf et al.^23^ developed composite sheets utilizing indigenous natural fibers (jute fiber) and a novel local epoxy resin (NOVO BOND EB) characterized by high strength. They conducted experimental and numerical investigations on the shear enhancement of RC beams wrapped in various configurations of jute fiber reinforced polymers (JFRP). The researchers determined that the maximum shear capacity of all reinforced specimens improved by 28% to 175% relative to the control sample. Bounjoum et al.^24^ performed an experimental study on concrete beams subjected to shear, reinforced with polymer matrix composites that included jute fibers via jacketing. The experiment’s results demonstrated a significant 69% enhancement in both stiffness and load capacity of the reinforced beam relative to its unreinforced counterpart. Mei et al.^25^ employed a novel category of large rupture strain FRP fabricated from eco-friendly and cost-effective polyethylene terephthalate (PET) fibers to investigate the shear strengthening of RC elements. The outcomes of three-point bending tests for fourteen shear-deficient RC beams strengthened by full-wrapping PET FRP were reported. The elevated rupture strain of PET FRP, over 7%, can prevent brittle failure of FRP and facilitate ductile shear failure during shear strengthening. Varma et al.^26^ conducted a study analyzing the effects of compressive axial stresses on concrete cylinders strengthened with jute and basalt fiber epoxy composites, assessing their strength and durability. Test results indicated that external confinement using hybrid FRP wraps, comprising both jute and basalt fibers, improved load bearing and energy absorption capacities by 63.64% and 287%, respectively.

Despite the extensive body of research on the strengthening of RC elements using FRP systems, existing studies have predominantly focused on synthetic fibers such as carbon and glass, with limited attention given to sustainable natural fiber alternatives. Moreover, most available investigations have examined either beams or columns independently, while direct comparative assessments of the effectiveness of fiber wrapping on both structural elements under consistent modeling assumptions remain scarce. In addition, although experimental studies dominate literature, comprehensive numerical investigations addressing the performance of RC beams and columns strengthened with both synthetic and natural fiber systems are still limited. This study addresses these gaps by conducting a FE-based comparative analysis of RC beams and columns strengthened with carbon, glass, and kenaf FRP laminates, thereby providing insight into the relative effectiveness of different fiber materials within a consistent analytical framework.

## Research objective and significance

Building on the identified research gaps, this study aims to provide a unified numerical framework for evaluating the effectiveness of FRP strengthening systems applied to RC beams and columns. Emphasis is placed on assessing the structural response of both element types under consistent modeling assumptions, with particular attention given to comparing sustainable natural fiber systems with conventional synthetic fiber alternatives. By examining the structural performance of kenaf alongside carbon and glass FRP systems, the study supports the development of more sustainable strengthening solutions that align with broader Sustainable Development Goals (SDGs) related to sustainable infrastructure and material efficiency.

To achieve these objectives, the present study focuses on the following key aspects:

- Developing FE models for RC beams and columns retrofitted with FRP laminates, which account for element types, mesh discretization, solution methodologies, and geometric and material nonlinearity, provides a cost-effective and rapid alternative to experimental research.
- Comparing the impact of wrapping RC beams and columns with various types of FRP on the overall performance of the RC element.
- Examining the impact of natural fibers as a sustainable alternative and comparing the findings with those derived from synthetic fibers.

### Development of a FE model for a RC beam

In this section, a detailed FE model was performed using ABAQUS FE analysis software^27^ to study the behavior of a simply supported RC beam. A FE modeling protocol is introduced, defining plentiful modeling considerations, which include: the solution techniques, the element type, the FE meshing, the boundary conditions, the modeling of different materials, and different components interactions in the model.

**Fig. 1:**
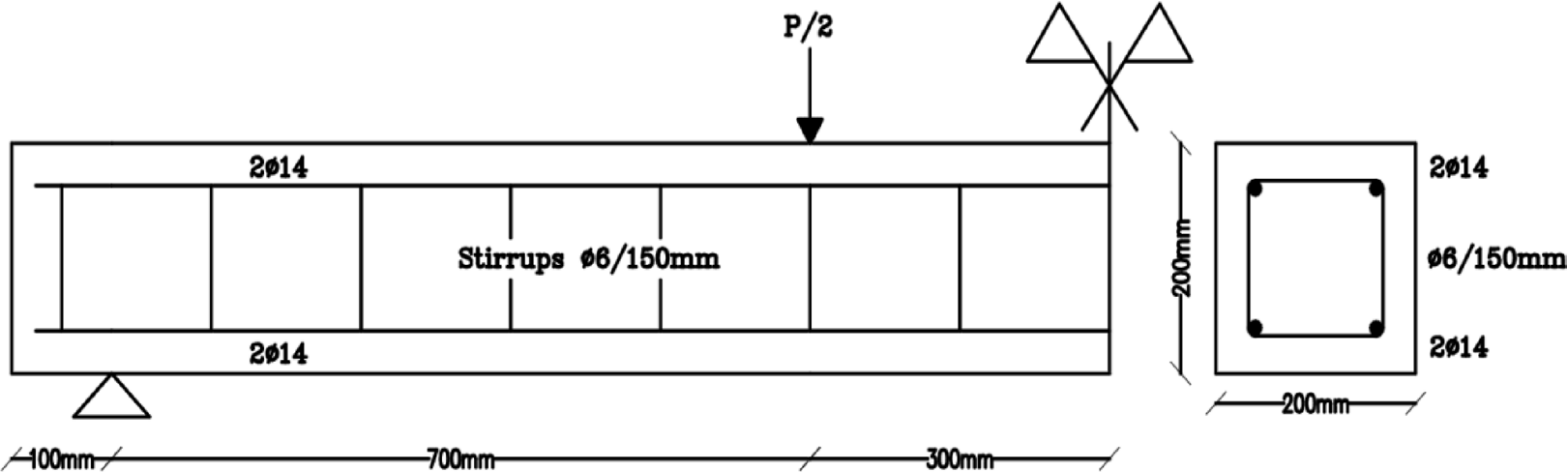
Experimentally tested Beam A1, Shehadeh et al.^28^.

**Fig. 2:**
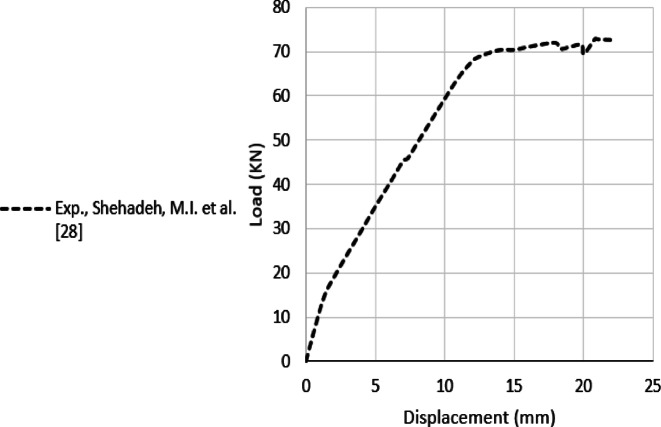
Load displacement curve of the tested Beam A1, Shehadeh et al.^28^.

The developed FE element model is verified against the results of Shehadeh et al^28^. Shehadeh et al.^28^ conducted a FE model for a simply supported RC beam and verified their model with the experimentally tested beam A1 shown in Fig. 1^28^. The experimental result of the tested beam A1 is shown in Fig. 2.

### Types of elements and mesh discretization

**Fig. 3:**
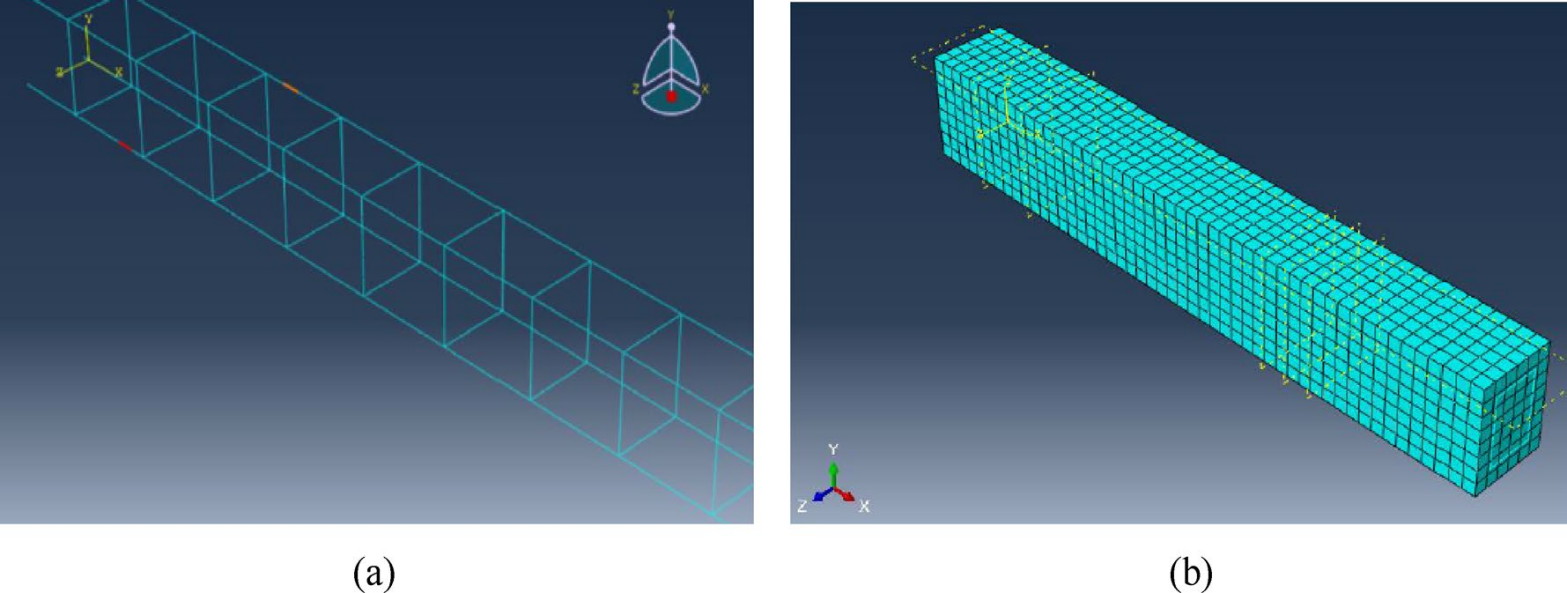
Generated Mesh in the FE model. (**a**) Meshing of the reinforcing bars & stirrups, (**b**) Meshing of the concrete beam.

The FE model is anticipated to integrate significant material and geometrical nonlinearities. Consequently, solid elements are preferable to shell elements as they accurately replicate anticipated physical behaviors and failure mechanisms. Accordingly, A 3D, 8-node linear element (C3D8R) with size of 50 mm is utilized to simulate the concrete beam. This element enhances computing efficiency while maintaining accuracy, ABAQUS 6.11 Documentation^27^. A two-node, three-dimensional truss element (T3D2) with a dimension of 50 mm is employed for steel reinforcement and steel stirrups. This element is utilized in two and three dimensions to represent slender, line-like structures that exclusively endure axial loading along the element, as per ABAQUS 6.11 Documentation^27^. The concrete beam was appropriately partitioned to produce a meshing outcome that is more structured and symmetrical, resulting in improved representation of stress gradients and enhanced accuracy of simulation results, Ismail^29,30^. Figure 3 illustrates the FE model, including the beam partitioning and the generated mesh.

### Solution procedures

Numerous solution strategies exist in ABAQUS that employ either implicit or explicit methodologies. The differences between implicit and explicit strategies can be easily demonstrated by examining the following formula that illustrates the implicit method:

1 $${U}^{n+1}=f\left({\ddot{U}}^{n+1},{\dot{U}}^{n+1},{U}^{n},\dots\dots\right)$$

The current node displacement, ${U}^{n+1}$, is dependent upon its time derivatives, which denote the unknown nodal velocity and acceleration. Thus, the implicit method resolves the equations by iteratively determining the nodal displacement, velocity, and acceleration. Conversely, the explicit technique can be described as follows:

2 $${U}^{n+1}=f\left({U}^{n},{\dot{U}}^{n},{{\ddot{U}}^{n},U}^{n-1},\dots\dots\right)$$

The current node displacement, ${U}^{n+1}$, is dependent upon the nodal displacement, velocity, and acceleration from the preceding time step. Since this arrangement is established, iteration and convergence testing are superfluous.

Two analysis approaches, Static-General and Dynamic implicit (Quasi-Static), were employed in the FE model to investigate the efficacy of each method. The comparison of the outcomes from both methods is to identify the most effective solution to the problem. Achieving convergence in Static-General analysis proved challenging unless a viscosity parameter was specified in the material specification of the concrete beam, as detailed in Sect.  2.4.2. Furthermore, automatic stabilisation with a stabilization-to-strain energy ratio of 0.05 is implemented in the analysis step to address convergence challenges. In the Dynamic Implicit (Quasi-Static) method, convergence was achieved without the necessity for a viscosity parameter or automatic stabilisation specification. The convergence is mostly due to fracture propagation and the progressive deterioration of the concrete beam.

### Loading application and boundary conditions

Loading is applied using the displacement-control method with a specified tabular amplitude, ensuring that displacement occurs solely in the direction of the load. Because solid elements are utilized, only axial degrees of freedom may be constrained, while the rotational movement is inactive within this model. Due to the symmetric test design and the applied load, only half of the tested beam is modelled. The diminishment of the model size enhances computing efficiency and facilitates the resolution of the models within a realistic timeframe, Abu-Hamd & Tawfik^31^. A plane of symmetry was established at the beam’s mid-span, constraining the beam’s move in the direction perpendicular to that plane. Symmetric constraints are imposed on a master node, with equation constraints applied to the slave nodes, which are to be constrained to the master node. Furthermore, an embedded constraint is incorporated into the model to integrate the reinforcing steel and stirrups within the concrete beam.

### Material properties

#### Steel

The reinforcing material properties utilized in this work were obtained from Shehadeh et al^28^. The existing FE model incorporates a linear elastic stress-strain relationship up to yielding, transitions to a perfectly plastic state between the elastic limit (${{\upepsilon}}_{\mathrm{y}}$) and the onset of strain hardening, and adheres to the constitutive law suggested by Gattesco^32^. for the strain-hardening segment:

3 $${\sigma}_{s}={f}_{sy}+{E}_{sh}\left({\epsilon}_{s}-{\epsilon}_{sh}\right).(1-{E}_{sh}.\frac{{\epsilon}_{s}-{\epsilon}_{sh}}{4\left({f}_{su}-{f}_{sy}\right)})$$

${f}_{sy}$ and ${f}_{su}$ denote the yield and ultimate tensile stresses of the steel component, whereas ${E}_{sh}$ and ${\epsilon}_{sh}$ represent the strain-hardening modulus and the strain at strain hardening of the steel component, respectively.

During the linear analysis phase, the reinforcement was modelled as an isotropic elastic material with a density of 7850 kg/m^2^, an elastic modulus of 210,000 MPa, and a Poisson’s ratio of 0.3. Furthermore, in the nonlinear analysis, the engineering stress (${\sigma}_{s}$) and the associated strain (${\epsilon}_{s}$) were converted to true stress (${\sigma}_{true}$) and true plastic strain (${\epsilon}_{true}^{pl}$) using the following equations:

4 $${\sigma}_{true}={\sigma}_{s}\left(1+{\epsilon}_{s}\right)$$

5 $${\epsilon}_{true}^{pl}=ln\left(1+{\epsilon}_{s}\right)-\frac{{\sigma}_{true}}{{E}_{s}}$$

Where ${\sigma}_{true}$represents true stress, ${\epsilon}_{true}^{pl}$ denotes true plastic strain, ${\sigma}_{s}$ signifies engineering stress, ${\epsilon}_{s}$ corresponds to the associated engineering strain, and ${E}_{s}$ indicates the initial elastic modulus of the engineering stress-strain curve.

**Fig. 4:**
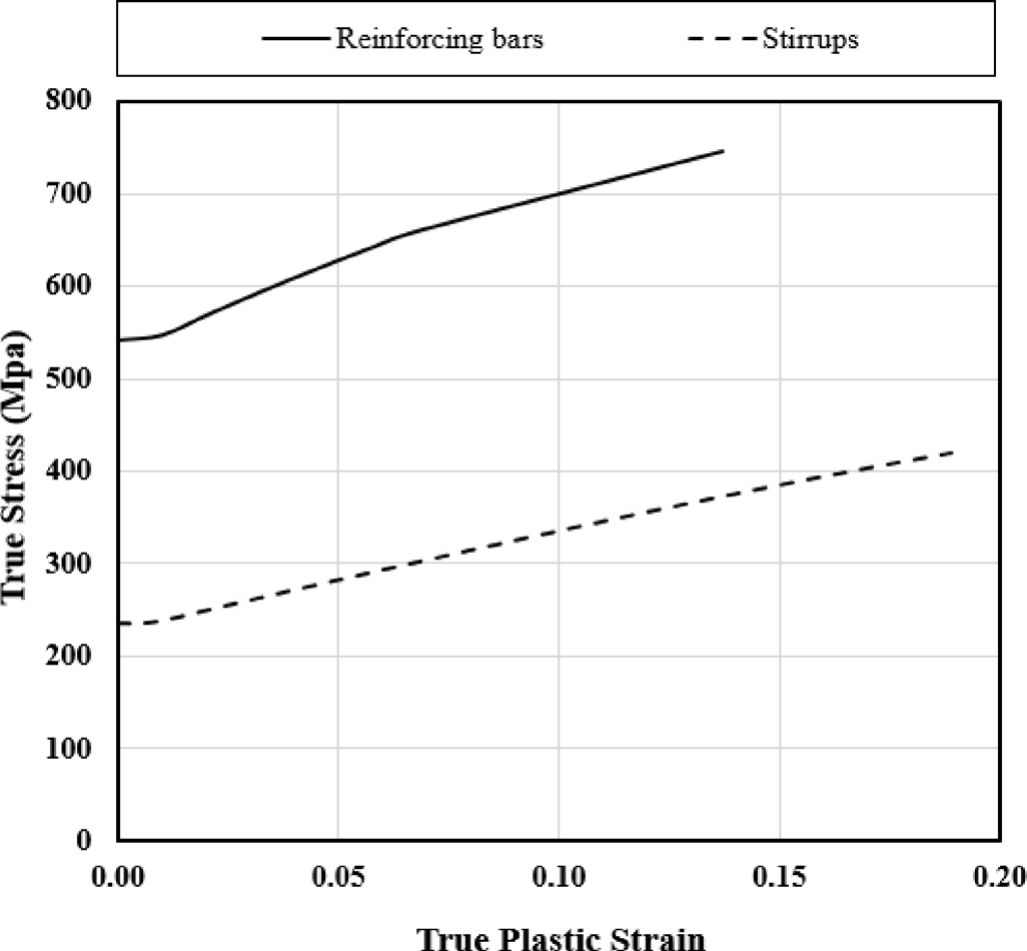
Reinforcement & stirrups stress-strain curves introduced in the FE model.

Figure 4 illustrates the stress-strain curves incorporated into ABAQUS^27^ for both the reinforcing bars and stirrups.

#### Concrete

**Fig. 5:**
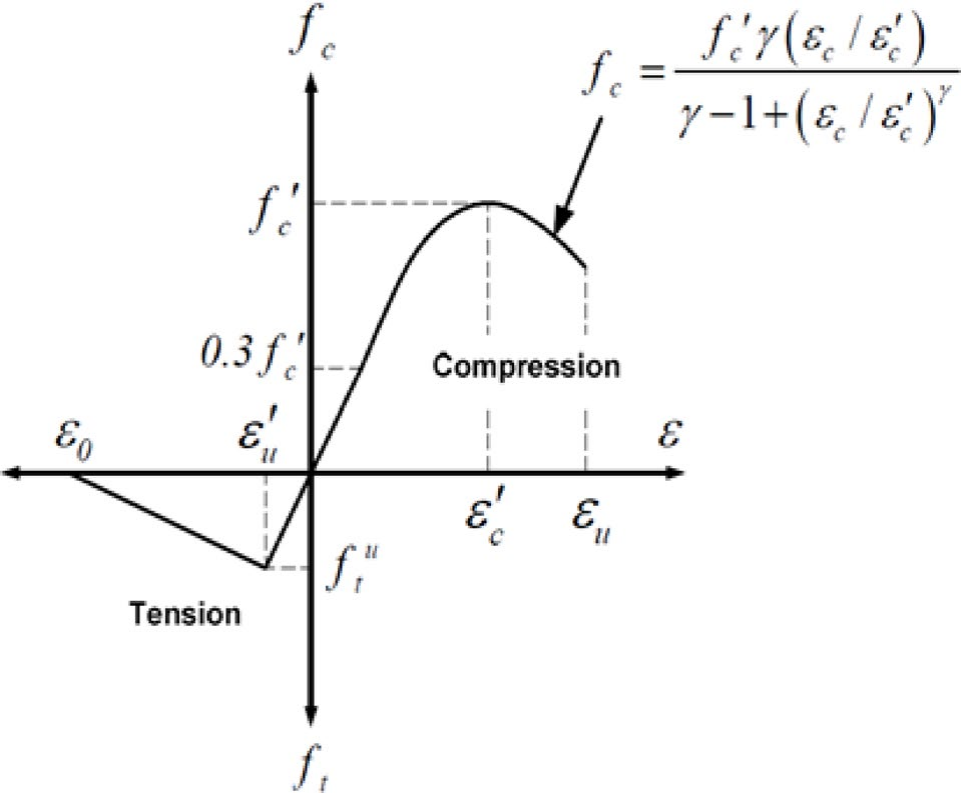
Uniaxial concrete stress strain curve, Carreira & Chu^35^.

The concrete material was simulated using the Plastic Damage model in ABAQUS, designed primarily for the analysis of RC structures. This model offers a realistic representation of concrete’s properties, while also possessing a general capability to model other quasi-brittle materials exhibiting similar failure modes, such as tensile cracking and compressive crushing, Tawfik & Abu-Hamd^33^. The Concrete Damage Plasticity (CDP) model utilizes plasticity theory and damage mechanics, integrating isotropic damaged elasticity with isotropic tensile and compressive plasticity to represent the inelastic behavior of materials. Furthermore, it incorporates damage characteristics to replicate the post-elastic behavior of the material under tension and compression. Consequently, the damage parameters *d*_*t*_ for tension and *d*_*c*_ for compression have been employed to illustrate the extent of degradation of the material’s original elasticity. The two parameters vary from zero to one, where zero signifies undamaged material and one denotes complete strength drop, according to ABAQUS 6.11 Documentation^27^. The material properties of concrete required for the FE model are classified into three categories: Elasticity, Plasticity, and Damage. The elasticity parameters are a modulus of elasticity $\mathrm{E}=25$ GPa and a Poisson’s ratio ${\upupsilon}=0.2$. The plasticity parameters are as follows: dilation angle $\Psi = 31^\circ$ (Hafezolghorani et al.^34^), eccentricity ${\upepsilon}=0.1$, stress ratio ${\mathrm{f}}_{\mathrm{b}0}/{\mathrm{f}}_{\mathrm{c}0}$ =1.16, shape factor $\mathrm{K}=0.66667$, and the viscosity parameter is $1\mathrm{E}-005$ throughout the Static-General procedure, while it is zero throughout the Quasi-Static procedure. Material models that demonstrate softening behavior and stiffness deterioration frequently result in significant convergence challenges in implicit analysis software, such as ABAQUS/Standard, ABAQUS 6.11 Documentation^27^. Certain convergence challenges can be mitigated through the application of a viscous regularisation scheme, which ensures that the tangent stiffness matrix of the softening material remains positive for adequately small-time increments. This is why the viscosity parameter is specified for the Static-General procedure rather than the Quasi-Static procedure. This work adopts the uniaxial stress-strain relationship for simulating the compressive behavior of concrete as proposed by Carreira & Chu^35^., illustrated in Fig. 5.

Where ${f}_{c}$ represents the compressive stress in concrete, ${\epsilon}_{c}$ denotes the compressive strain in concrete, ${f}_{c}^{{\prime}}$signifies the cylinder compressive strength of concrete, ${\epsilon}_{c}^{{\prime}}$ corresponds to the strain associated with ${f}_{c}^{{\prime}}$, and ${\gamma}_{c}$ is defined as:

6 $${\gamma}_{c}={\left|\frac{{f}_{c}^{{\prime}}}{32.4}\right|}^{3}+1.55$$

**Fig. 6:**
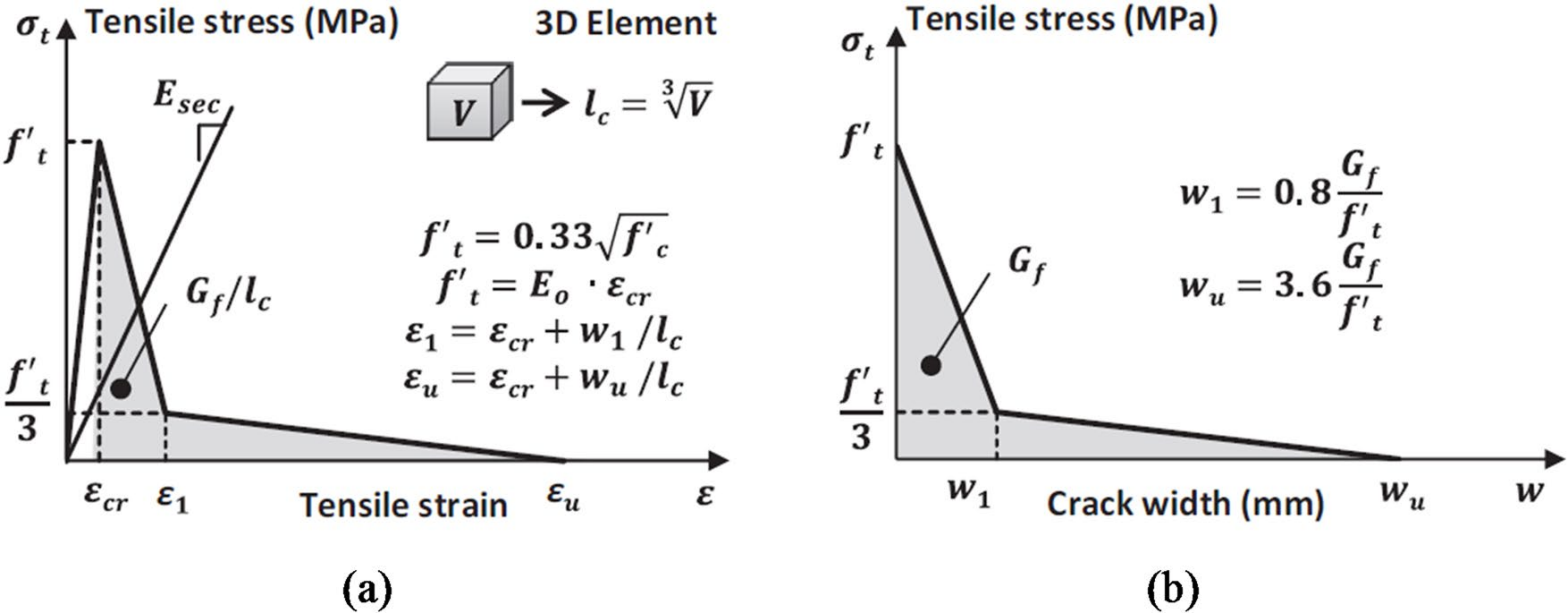
(**a**) Uniaxial tensile stress strain relationship for concrete, (**b**) Uniaxial tensile stress-crack width relationship for concrete, Genikomsou & Polak^36^.

According to Genikomsou & Polak^36^, the uniaxial stress-strain behavior of concrete is linear elastic until it reaches its tensile strength, ${\mathrm{f}}_{\mathrm{t}}^{{\prime}}$. Following cracking, the descending branch is represented by a softening process that concludes at a tensile strain ${{\upepsilon}}_{\mathrm{u}}$, at which point there is no residual tensile strength, as illustrated in Fig. 6a.

The brittle behavior of concrete is typically defined by a stress-crack displacement response rather than a stress-strain relationship. The stress-crack displacement relationship may be characterized by various models: linear, bilinear, or exponential tension softening response. This study employed a bilinear stiffening response, calculated as depicted in Fig. 6b, where ${\mathrm{f}}_{\mathrm{t}}^{{\prime}}$ indicates the maximum tensile strength and ${\mathrm{G}}_{\mathrm{f}}$ signifies the fracture energy of concrete, which corresponds to the area beneath the tensile stress-crack displacement curve.

The fracture energy ${\mathrm{G}}_{\mathrm{f}}$ is contingent upon the quality of the concrete and the size of the aggregate and can be derived from Eq. (7) of the CEB-FIP Model Code 90^37^.

7 $${G}_{f}={G}_{fo}({{f}_{cm}/{f}_{cmo})}^{0.7}N/mm$$

Where ${\mathrm{f}}_{\mathrm{c}\mathrm{m}\mathrm{o}}$equals $10\mathrm{M}\mathrm{P}\mathrm{a}$ and ${\mathrm{G}}_{\mathrm{f}\mathrm{o}}$ represents the base fracture energy contingent upon the maximum aggregate size, ${\mathrm{d}}_{\mathrm{m}\mathrm{a}\mathrm{x}}$. The basic fracture energy ${\mathrm{G}}_{\mathrm{f}\mathrm{o}}$ is $0.026\mathrm{N}/\mathrm{m}\mathrm{m}$ for a maximum aggregate size ${\mathrm{d}}_{\mathrm{m}\mathrm{a}\mathrm{x}}$ of $10\mathrm{m}\mathrm{m}$^37^. As per^37^, ${\mathrm{f}}_{\mathrm{c}\mathrm{m}}$ denotes the mean compressive strength of concrete, and its correlation with the characteristic value, ${\mathrm{f}}_{\mathrm{c}}^{{\prime}}$, is:

8 $${f}_{cm}={f}_{c}^{{\prime}}+8MPa$$

To minimize fracture localization, tensile strains were employed, defined as the ratio of cracking displacement ($$w)$$ to the characteristic length of the element ($${l}_{c})$$. The characteristic length for 3D elements is defined as the cube root of the element’s volume. The designated characteristic length ($${l}_{c})$$ in the subsequent simulations was 46.3 mm.

Damage was incorporated into the CDP model, wherein concrete damage was presumed to manifest within the softening range for both compression and tension. The damage occurred after attaining the peak load associated with the strain levels, ${\epsilon}_{c}^{{\prime}}$ and ${\epsilon}_{cr}$, in compression and tension, respectively.

Compressive plastic hardening strain ${\epsilon}_{c}^{pl,h}$ is essential in establishing the correlation between the damage parameters and compressive strength as follows:

9 $${\sigma}_{c}=\left(1-{d}_{c}\right){E}_{0}\left({\epsilon}_{c}-{\epsilon}_{c}^{pl,h}\right)$$

10 $$\left\{\begin{array}{c}{\epsilon}_{c}^{in,h}={\epsilon}_{c}-\frac{{\sigma}_{c}}{{E}_{0}}\\{\epsilon}_{c}^{pl,h}={\epsilon}_{c}-\frac{{\sigma}_{c}}{{E}_{0}}\left(\frac{1}{1-{d}_{c}}\right)\end{array}\right.$$

11 $${\epsilon}_{c}^{pl,h}={\epsilon}_{c}^{in,h}-\frac{{d}_{c}}{\left(1-{d}_{c}\right)}\frac{{\sigma}_{c}}{{E}_{0}}$$

Compressive damage *dc* is contingent upon the compressive inelastic strain ${\epsilon}_{c}^{in,h}$. Given that *dc* escalates with the increase of the inelastic strain ${\epsilon}_{c}^{in,h}$, *dc* can be expressed by the subsequent formula:

12 $${d}_{c}=1-\frac{{\sigma}_{c}}{{\sigma}_{cu}}$$

The tensile plastic hardening strain ${\epsilon}_{t}^{pl,h}$ is defined as follows:

13 $${\sigma}_{t}=\left(1-{d}_{t}\right){E}_{0}\left({\epsilon}_{t}-{\epsilon}_{t}^{pl,h}\right)$$

14 $$\left\{\begin{array}{c}{\epsilon}_{t}^{ck,h}={\epsilon}_{t}-\frac{{\sigma}_{t}}{{E}_{0}}\\{\epsilon}_{t}^{pl,h}={\epsilon}_{t}-\frac{{\sigma}_{t}}{{E}_{0}}\left(\frac{1}{1-{d}_{t}}\right)\end{array}\right.$$

15 $${\epsilon}_{t}^{pl,h}={\epsilon}_{t}^{ck,h}-\frac{{d}_{t}}{\left(1-{d}_{t}\right)}\frac{{\sigma}_{t}}{{E}_{0}}$$

Tensile damage *d*_*c*_ is contingent upon the tensile cracking strain ${\epsilon}_{t}^{ck,h}$. Given that *d*_*c*_ escalates with an increase in the cracking strain ${\epsilon}_{t}^{ck,h}$, *d*_*c*_ can be expressed using the subsequent formula:

16 $${d}_{t}=1-\frac{{\sigma}_{t}}{{\sigma}_{t0}}$$

**Fig. 7:**
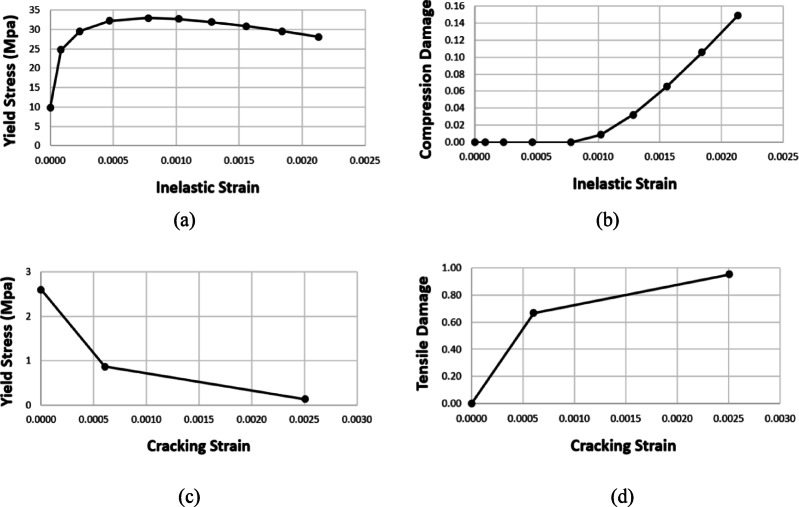
Curves of concrete employed in the FE model: (**a**) Yield stress & Inelastic strain, (**b**) Compressive damage & Inelastic strain, (**c**) Yield stress & Cracking strain, (**d**) Tensile damage & Cracking strain.

According to the aforementioned stress-strain curves and equations, strain softening curves, and damage evolution curves illustrated in Fig. 7 were integrated into the FE model, as they are crucial for identifying plasticity and damage.

### FE model verification

**Fig. 8:**
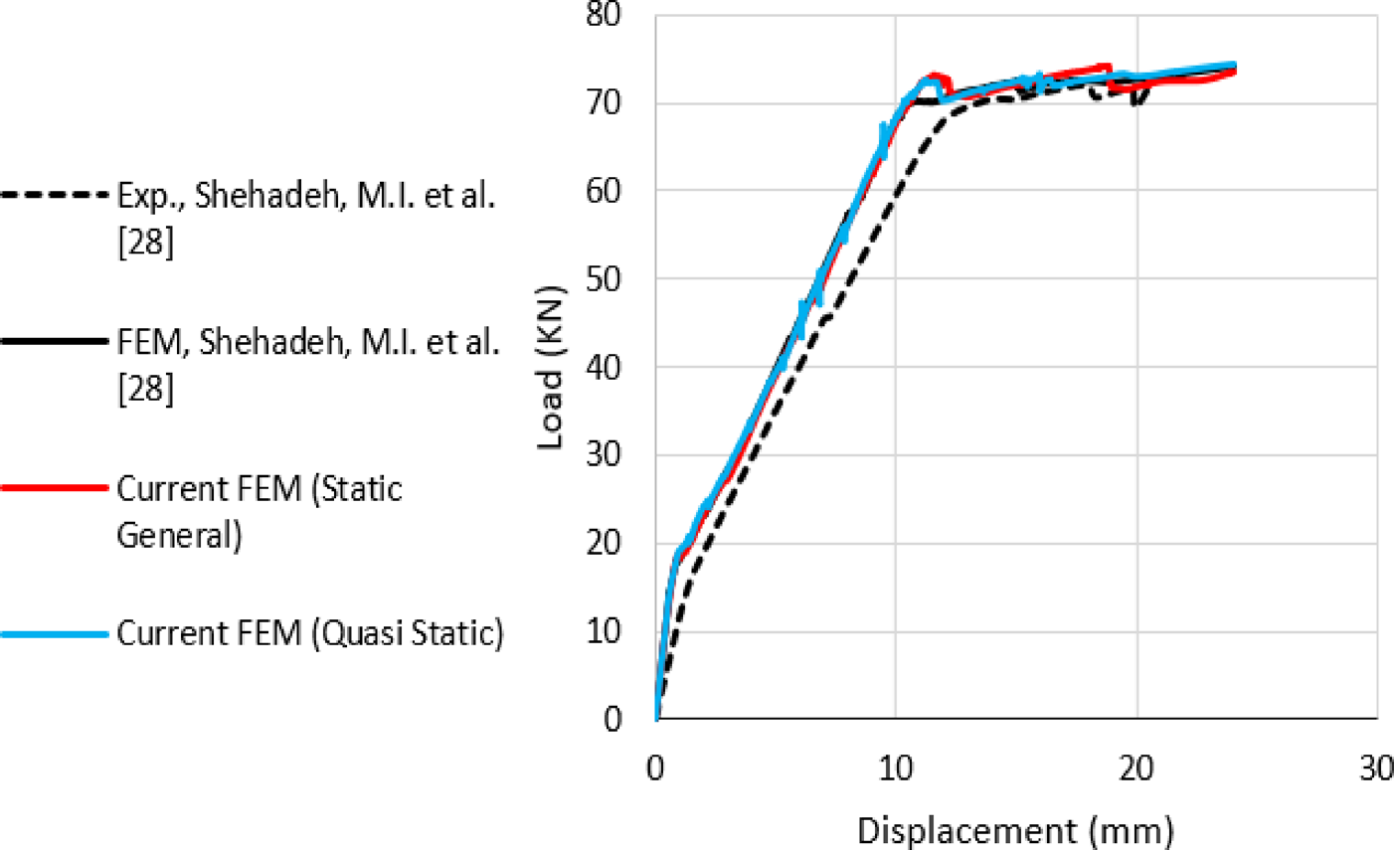
Comparison of experimental and computational findings for the current FE models and the results of Shehadeh et al^28^.

The FE models are validated against the findings of Shehadeh et al.^28^, as presented in Fig. 8, exhibiting a strong correlation in stiffness and strength analysis. Specifically, the ultimate loads reported by Shehadeh et al. ^28^ through experimental and numerical methods are 72.5 kN and 73.2 kN, respectively, while the present study yields ultimate loads of 73.8 kN and 73.4 kN using the Static-General and Quasi-Static solvers, respectively.

By comparing the ultimate load values, it could be found that the difference between the current FE model’s strength prediction and the results reported by Shehadeh et al.^28^ didn’t exceed 1.8%. egarding the solution techniques implemented in this study, Table 1 summarized the differences between the static General and the Quasi Static procedures.

**Table 1:** Comparison of static general and Quasi static procedures.

Analysis procedure	Viscosity parameter	Stabilization ratio	Total number of increments	Solution time (minutes)
Static general	1E-005	0.05	602	5
Quasi static	0	0	487	3

**Fig. 9:**
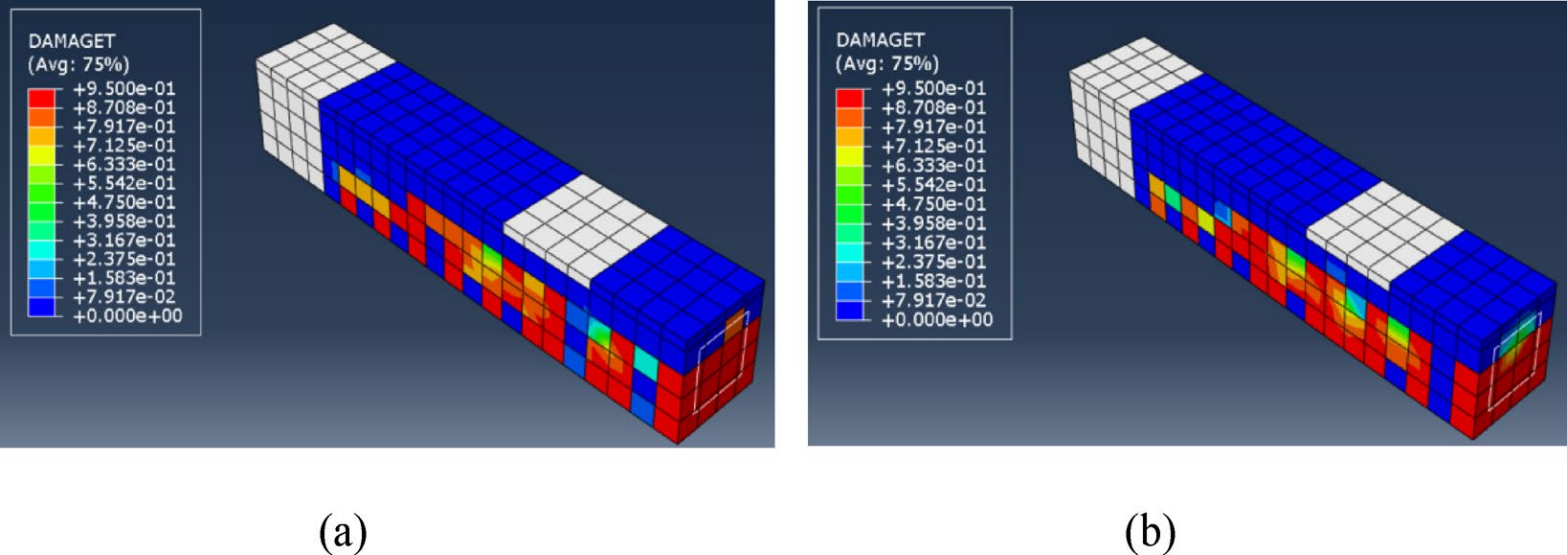
Tension Damage (**a**) Static General, (**b**) Quasi Static. (**a**) Tension Damage – Static General, (**b**) Tension Damage – Quasi Static.

**Fig. 10:**
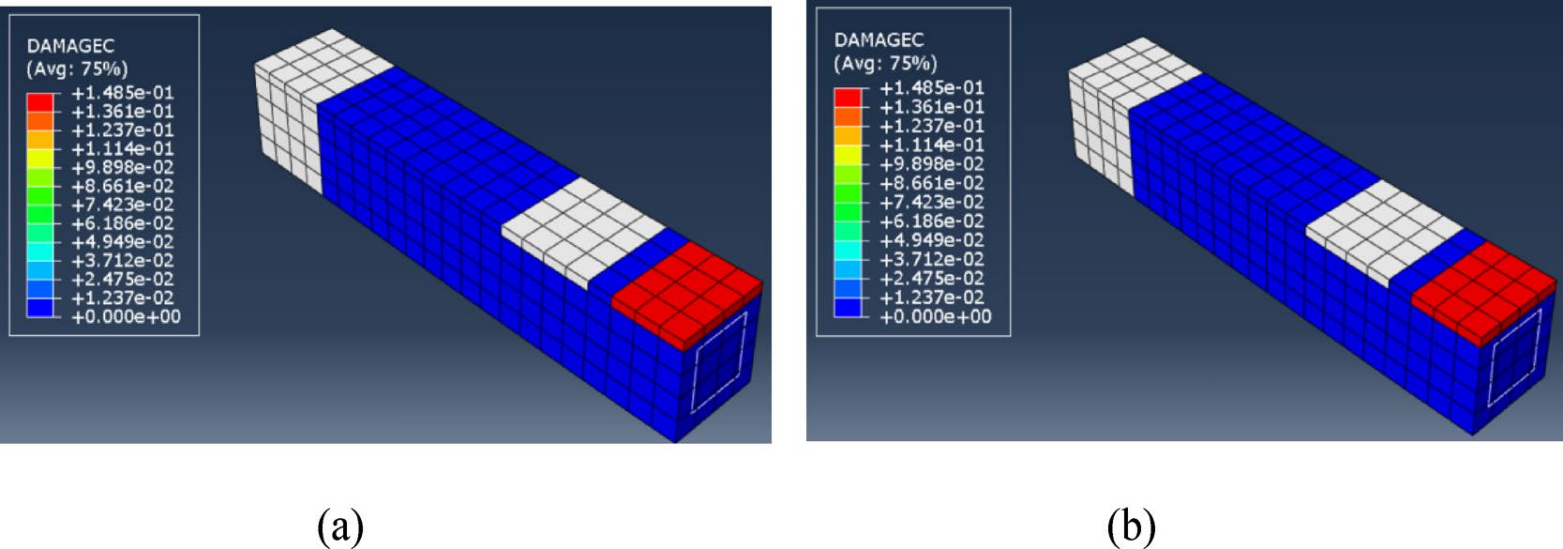
Compression Damage (**a**) Static General, (**b**) Quasi Static. (**a**) Compression Damage – Static General, (**b**) Compression Damage – Quasi Static.

The above table indicates that the Quasi Static analysis approach exceeds the Static General approach regarding convergence concerns, as it does not require a viscosity parameter or stabilization ratio. The total number of increments in the Quasi-Static analysis was reduced, and the solution time for the Quasi-Static analysis was 60% less than that of the Static General procedure. The modes of failure indicated by the current FE models are shown in Figs. 9 and 10 for the tension damage and the compression damage, respectively, for the above-mentioned solution procedures.

### Strengthening RC elements with CFRP, GFRP and KFRP laminates

Based on the previous validation, a comparative analysis was conducted to assess the influence of fiber wraps on the structural performance of RC beams and columns, focusing on the degree to which fiber wrapping enhances the behavior of these components and determining which element exhibits greater enhancement. The study also examines the impact of several fiber materials, including CFRP and GFRP, to identify the most effective type for structural strengthening. The research evaluates the viability of sustainable, green fibers by utilizing kenaf fibers and comparing their effectiveness with that of conventional fiber materials. Kenaf fiber is a natural bast fiber obtained from the Hibiscus cannabinus plant and is widely available due to its rapid growth and renewability. From an engineering perspective, kenaf fibers are characterized by low density and favorable specific tensile strength and stiffness, making them suitable for use in FRP composites. Previous studies have reported that the mechanical performance of KFRP can be comparable to that of conventional synthetic fibers, such as glass fibers, while offering advantages in terms of reduced weight and environmental impact, Saba et al.^38^. Moreover, experimental and numerical investigations have demonstrated that KFRP laminates can effectively enhance the flexural performance of RC members, providing measurable improvements in load-carrying capacity and deformation control compared to unstrengthened elements, Nwankwo & Ede^22^. Owing to its renewable nature, biodegradability, and lower carbon footprint, kenaf fiber has gained increasing attention in construction-related composite applications, Mahir et al^39^. Accordingly, KFRP was selected in this study to represent a sustainable strengthening alternative and to enable a direct comparison with conventional CFRP and GFRP systems under identical modeling conditions, thereby isolating the influence of fiber material type on structural performance.

Utilizing the methodology outlined in Sect.  2 for FE model development, eight models, four beams and four columns, were constructed to evaluate their structural behavior with the specified fiber laminates. Each structural element included a control specimen alongside three specimens strengthened with KFRP, GFRP, and CFRP, respectively.

### Fiber laminates modeling

The material characteristics of fiber laminates were simulated using the Hashin damage model, Szpoganicz et al.^40^, within ABAQUS, which forecasts damage initiation and models damage evolution for elastic-brittle materials exhibiting anisotropic behavior. The model is mostly designed for application with fiber-reinforced materials, as they generally exhibit this behavior. Damage is characterized by the deterioration of material stiffness. It is crucial in the analysis of fiber-reinforced composite materials. Numerous materials display elastic-brittle behavior, wherein damage occurs without considerable plastic deformation. Therefore, plasticity may be disregarded when modelling the behavior of such materials. The Hashin damage model, as proposed by Muzaqih et al.^41^, necessitates the definition of the material’s undamaged response, which must exhibit linear elasticity, a criterion for damage initiation, and a response for damage evolution, including the selection of element removal. The anisotropic elastic properties of fiber laminates can be modelled using the “LAMINA” material type. Damage initiation denotes the commencement of deterioration at a specific material location. The damage initiation criteria for fiber-reinforced composites, according to Hashin’s theory, encompasses four distinct damage initiation mechanisms: fiber rupture in tension, fiber buckling and kinking in compression, matrix cracking under transverse tension and shearing, and matrix crushing under transverse compression and shearing. Damage may ensue when the rupture energy for any of the four criteria meets or above its maximum threshold.

**Fig. 11:**
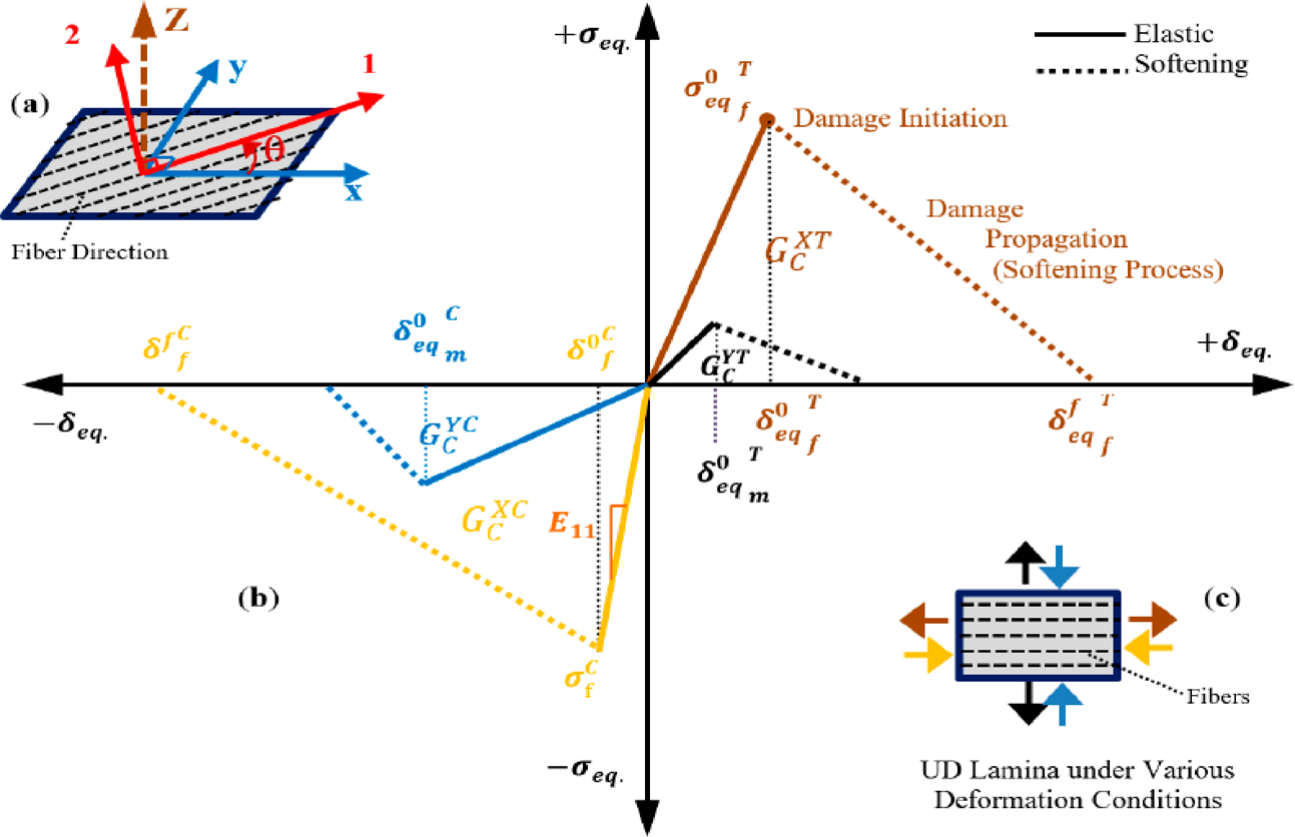
(**a**) Local (1–2) and global (x-y) axes of an angle lamina, (**b**) bilinear stress-strain behavior of FRP lamina in orthogonal axes for various failure modes, (**c**) each colored curve corresponds to the loading as shown by the same-colored arrows in the inset figure Koloor et al^42^.

Figure 11b illustrates the uniaxial behavior of unidirectional fiber composite lamina along the orthogonal axes (1–2 axis, Fig. 11a) concerning elastic-damage behavior under both tension and compression. The four bilinear elastic softening curves illustrate the equivalent stress-displacement response of composite lamina under various failure modes, including matrix cracking, crushing, fiber breaking, and buckling, as indicated by the load arrows of unidirectional lamina (Fig. 11b). In an angled lamina subjected to global loading (x–y axis, Fig. 11a), the global deformations are translated into local deformations to calculate the effective stress parameters.

The orthotropic definition of unidirectional FRP laminates follows the formulation described by Kezmane et al^43^. The unidirectional composite strips possess three orthogonal property planes: planes $12=\mathrm{x}\mathrm{y},13=\mathrm{x}\mathrm{z},\mathrm{a}\mathrm{n}\mathrm{d}23=\mathrm{y}\mathrm{z}.$ The coordinate axes $1,2,$ and $3$ denote the principal coordinates of the material, with $1$ being the fiber direction ($\mathrm{x}-\mathrm{a}\mathrm{x}\mathrm{i}\mathrm{s}$) and $2$ and $3$ ($\mathrm{y}\mathrm{a}\mathrm{n}\mathrm{d}\mathrm{z},$respectively) being orthogonal to $1$. In this instance, the material parameters in directions $2$ and $3$ are identical (E_22_ = E_33_, ν_23_ = ν_32_), hence classifying the material as orthotropic. Under stress conditions in the plane of a shell element, just the parameters E_11_, E_22_, ν_12_, G_12_, G_13_, and G_23_ are necessary to characterize the orthotropic material. The Poisson’s ratio ν_21_ is expressed as ν_21_=(E_22_/E_11_) ν_12_. The elastic properties of the material can be articulated as follows:

17 $$\left(\genfrac{}{}{0pt}{}{{\epsilon}_{1}}{\begin{array}{c}{\epsilon}_{2}\\{\gamma}_{12}\end{array}}\right)=\left[\begin{array}{ccc}1/{E}_{11}&{-\upsilon}_{12}/{E}_{11}&0\\{-\upsilon}_{12}/{E}_{11}&1/{E}_{22}&0\\0&0&1/{G}_{12}\end{array}\right]\left(\genfrac{}{}{0pt}{}{{\sigma}_{1}}{\begin{array}{c}{\sigma}_{2}\\{\tau}_{12}\end{array}}\right)$$

The parameters can be ascertained by Eqs. (18)–(24) utilizing the mixture law established by Piggott^44^.

18 $${E}_{11}={V}_{f}{E}_{f}+{E}_{m}(1-{V}_{f})$$

19 $${E}_{22}={E}_{33}=\frac{{E}_{m}{E}_{f}}{{E}_{m}{V}_{f}+(1-{V}_{f}){E}_{f}}$$

20 $${\upsilon}_{12}={\upsilon}_{13}={\upsilon}_{f}{V}_{f}+{\upsilon}_{m}(1-{V}_{f})$$

21 $${\upsilon}_{23}={E}_{22}\left[{V}_{f}\frac{{\upsilon}_{f}}{{E}_{f}}+(1-{\upsilon}_{f})\frac{{\upsilon}_{m}}{{E}_{m}}\right]$$

22 $${G}_{13}={G}_{12}=\frac{{G}_{m}{G}_{fb}}{{G}_{m}{V}_{f}+(1-{V}_{f}){G}_{fb}}$$

23 $${G}_{23}=\frac{{E}_{22}}{2(1+{\upsilon}_{23})}$$

24 $${G}_{m}=\frac{{E}_{m}}{2(1+{\upsilon}_{m})};{G}_{fb}=\frac{{E}_{f}}{2(1+{\upsilon}_{f})}$$

where E_11_, E_22_ and E_33_ denote the modulus of elasticity in x, y, and z directions, respectively (MPa); G_13_, G_12_ and G_23_ represent the shear modulus in the xz, xy, and yz directions (MPa); ν_13_, ν_12_ and ν_23_ indicate the Poisson’s ratios in xz, xy, and yz directions, respectively; E_f_, ν_f_, G_fb_, and E_m_, ν_m_, G_m_ refer to the elastic modulus (MPa), Poisson’s ratios, and shear modulus (MPa) of the fibers and matrix, respectively; V_f_ signifies the volume of the fibers in the composite. The elastic and strength characteristics of GFRP and CFRP composite laminates utilized in the FE simulations were acquired by standardized testing^45–48^, whereas the fracture energy values were derived from the properties of analogous materials documented in the literature^49–51^, as presented in Table 2.

**Table 2:** Elastic and damage properties of unidirectional GFRP and CFRP composite laminas.

lamina constants			Constitutive damage model parameters of lamina			
	GFRP	CFRP			GFRP	CFRP
${E}_{11,}GPa$	36.9	105.5	Longitudinal tensile strength, MPa	${X}_{T}$	820	1340
${E}_{22,}GPa$	10	7.2	Longitudinal compressive strength, MPa	${X}_{C}$	500	1192
${E}_{33,}GPa$	10	7.2	Transverse tensile strength, MPa	${Y}_{T}$	80.6	19.6
${G}_{12,}GPa$	3.3	3.4	Transverse compressive strength, MPa	${Y}_{C}$	322	92.3
${G}_{13,}GPa$	3.3	3.4	Longitudinal shear strength, MPa	${S}_{L}$	54.5	51
${G}_{23,}GPa$	3.6	2.52	Transverse shear strength, MPa	${S}_{T}$	161.2	23
${\nu}_{12}$	0.32	0.34	Longitudinal tensile fracture energy, N/mm	${G}_{XT}$	32	48.4
${\nu}_{13}$	0.32	0.34	Longitudinal comp. fracture energy, N/mm	${G}_{XC}$	20	60.3
${\nu}_{23}$	0.44	0.378	Transverse tensile fracture energy, N/mm	${G}_{YT}$	4.5	4.5
			Transverse comp. fracture energy, N/mm	${G}_{YC}$	4.5	8.5

For KFRP laminate, the material was assigned orthotropic properties proposed by Nwankwo & Ede^22^. and listed in Table 3.

**Table 3:** KFRP laminate orthotropic properties.

KFRP lamina constants					
${E}_{11,}MPa$	13,918	${G}_{12,}MPa$	5061	${\nu}_{12}$	0.375
${E}_{22,}MPa$	4566	${G}_{13,}MPa$	5061	${\nu}_{13}$	0.375
${E}_{33,}MPa$	4566	${G}_{23,}MPa$	2033	${\nu}_{23}$	0.123

**Fig. 12:**
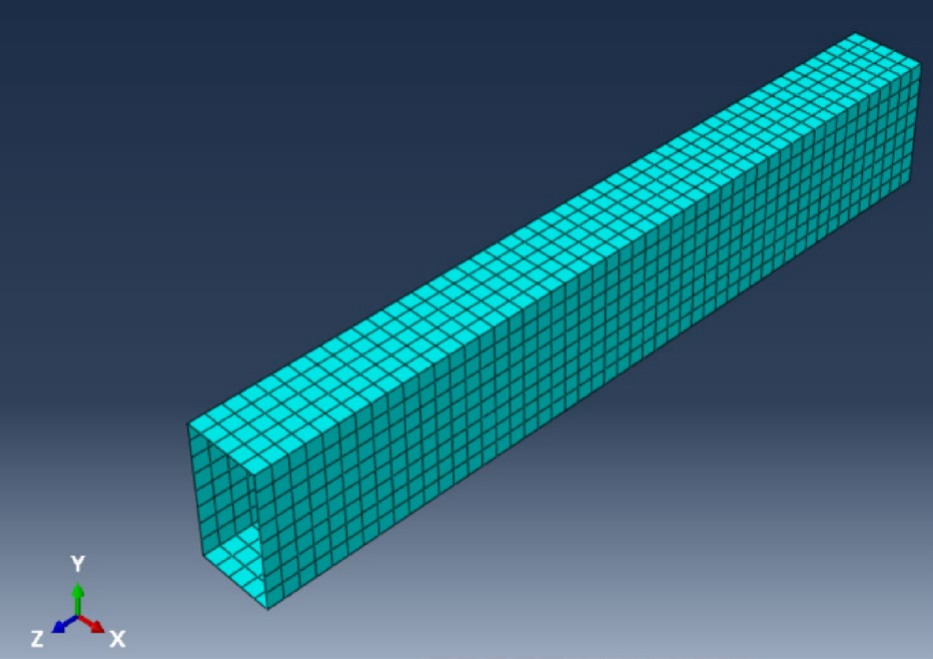
Meshing of the fiber wraps.

FRP sheets were modelled using a 4-node shell element (S4R) with a size of 40 mm. The S4R element accommodates both linear and nonlinear behavior, accurately modelling the anisotropic characteristics of FRP while decreasing computational expenses and mitigating shear locking in slender structures, as noted in ABAQUS 6.11 Documentation^27^. The FRP laminates were modeled with a constant stacking sequence and a constant ply thickness of 0.146 mm per layer (eight plies, total laminate thickness ≈ 1.17 mm) across all models, such that the fiber type was the sole variable investigated. A perfect bond between the RC element and the laminates is assumed through the application of a Tie constraint. Figure 12 illustrates the fiber wrap along with the generated mesh.

### Strengthening RC Beams with CFRP, GFRP and KFRP laminates

**Fig. 13:**
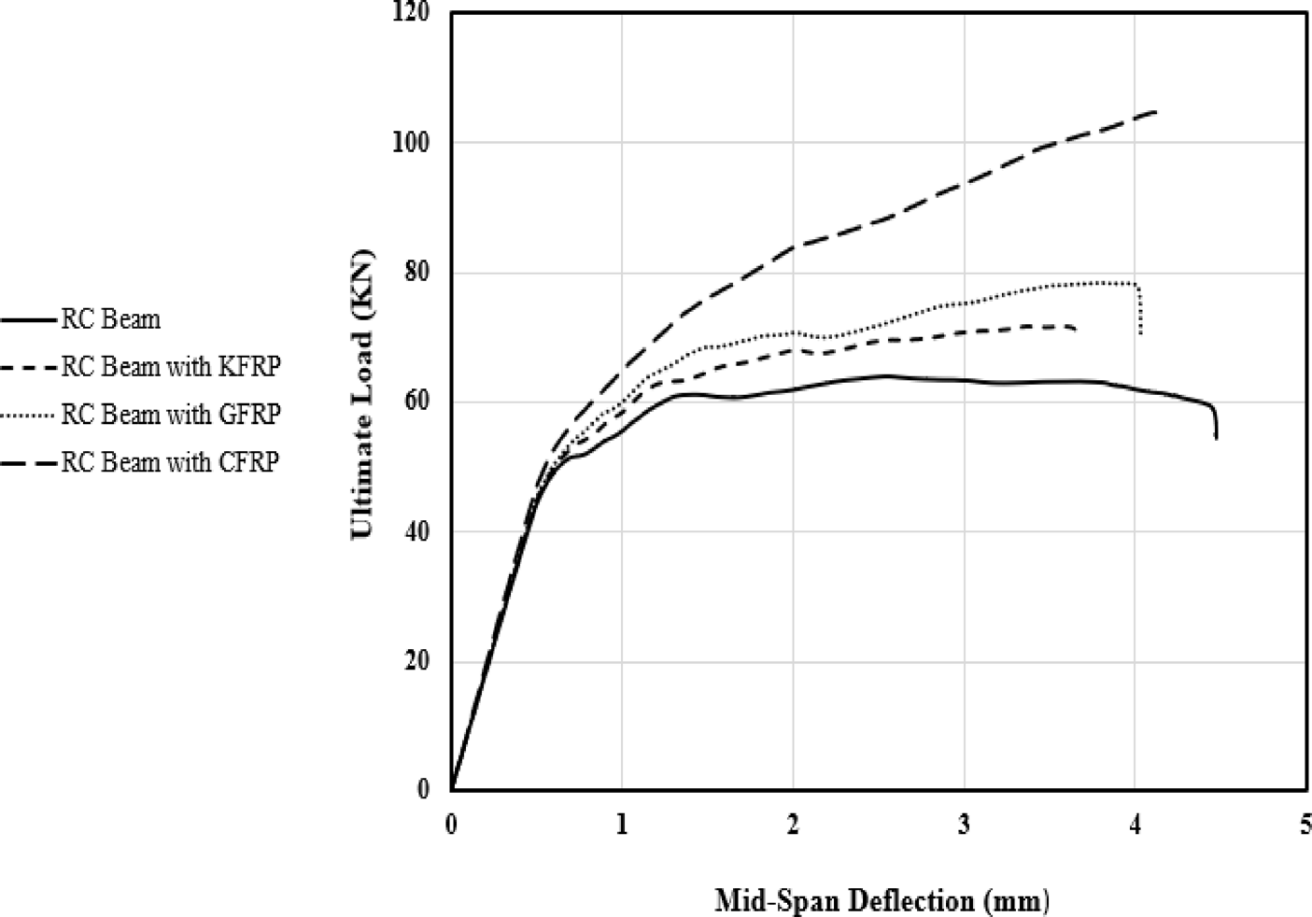
FE load displacement relationships - Influence of fiber wrapping on RC beams.

This section examines the impact of wrapping RC beams with GFRP, CFRP, and KFRP by modelling three wrapped beams, each utilizing a different fiber type, and comparing their structural performance with that of an unwrapped RC beam. Figure 13 illustrates the load displacement relationships derived for the investigated beams, highlighting the effects of various fiber wrapping on the beams’ stiffness, strength, and ductility.

Figure 13 illustrates that wrapping RC beams with various types of fiber laminates significantly enhances their performance during the plastic stage (the onset of yielding and the ultimate load capacity), while exerting negligible influence on the initial stiffness, which is contingent upon the modulus of elasticity of concrete material. Fiber wrapping notably enhanced the ultimate load across various fiber laminates. The control beam had an ultimate load of 63 kN, while the incorporation of KFRP, GFRP, and CFRP increased the values to 71.5 kN, 78.4 kN, and 104.6 kN, respectively. Additionally, the midspan deflection measured 4.3 mm for the control beam, decreasing to 3.4 mm, 3.8 mm, and 4.0 mm for KFRP, GFRP, and CFRP, respectively.

The ductility of the beams can be quantified using the toughness ratio (TR), as reported by Dabaon^52^.

25 $$TR={T}_{u}/{T}_{e}$$

${\mathrm{T}}_{\mathrm{u}}$ represents the area beneath the ultimate curve of the load-deflection relationship, while ${\mathrm{T}}_{\mathrm{e}}$ denotes the area beneath the elastic curve.

The ductility of the beams significantly improved with fiber wrapping, as reflected in the toughness ratios. While the control beam exhibited a toughness ratio of 16.62, the application of KFRP, GFRP, and CFRP increased the ratios to 19.85, 23.74, and 28.6, respectively.

**Fig. 14:**
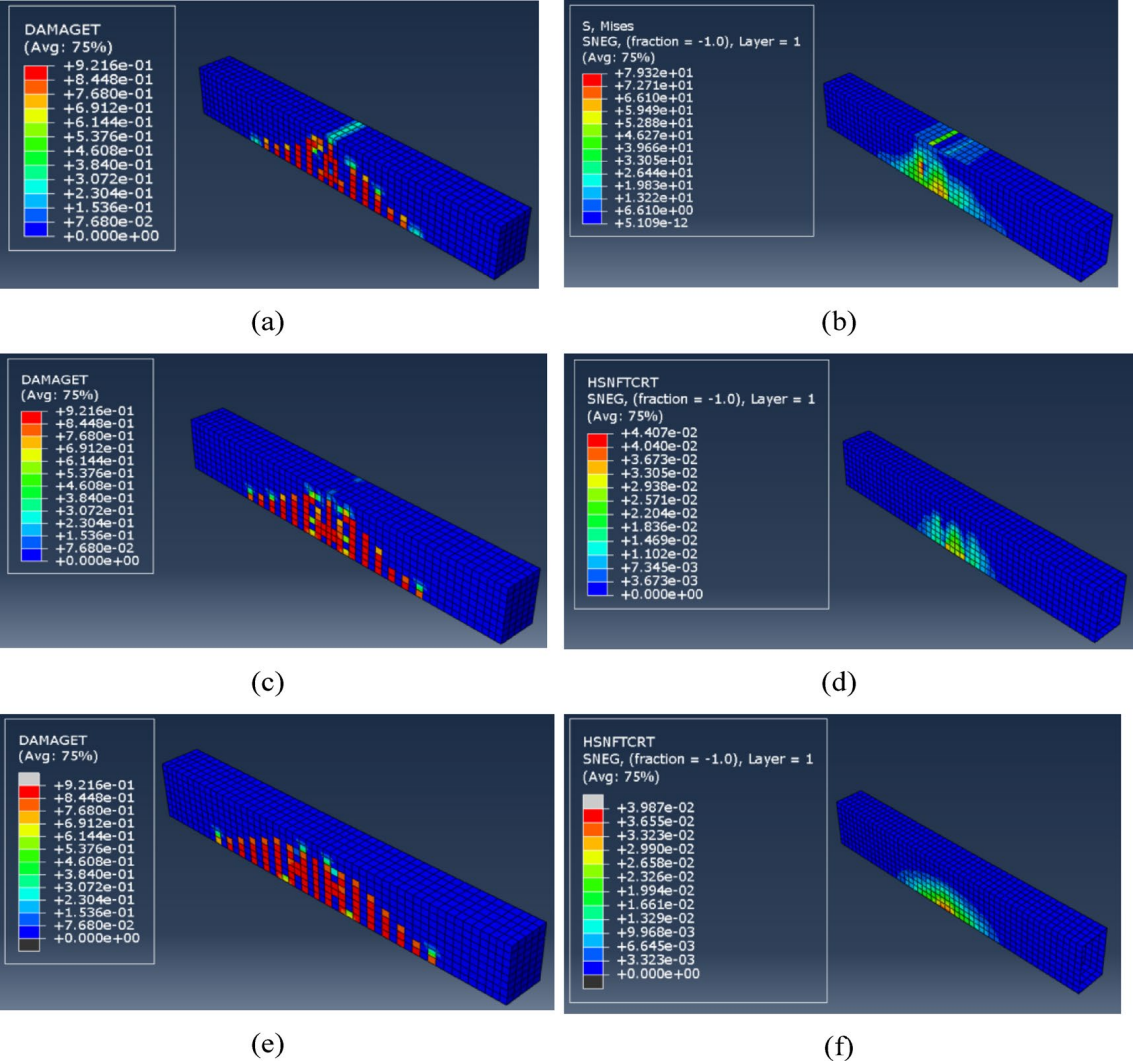
(**a**) Concrete tension damage for beam with KFRP, (**b**) Stress in KFRP, (**c**) Concrete tension damage for beam with GFRP, (**d**) Tension damage of GFRP, (**e**) Concrete tension damage for beam with CFRP, (**f**) Tension damage of CFRP.

These results align with Al Shboul et al.^11^, who introduced a paradigm shift from conventional thinking that FRP strengthening solely enhances ultimate capacity; they proposed a novel strengthening technique that significantly improves ductility and serviceability. The failure modes of the beams can be illustrated by the damage of the concrete material and the fiber wrapping. Figure 14 illustrates the failure of the wrapped beams utilizing the three types of fibers under investigation.

### Strengthening RC Columns with CFRP, GFRP and KFRP laminates

**Fig. 15:**
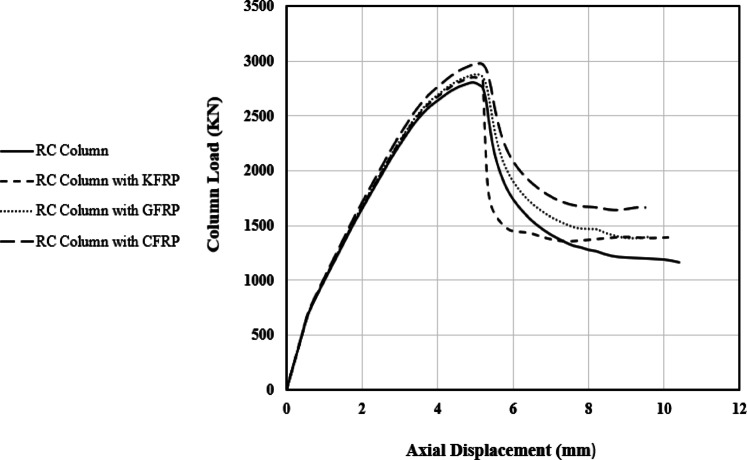
FE load displacement relationships - Influence of fiber wrapping on RC columns.

This section examines the impact of wrapping RC short columns with GFRP, CFRP, and KFRP. Three wrapped columns, each utilizing a different fiber type, are modeled under pure axial static load, and their structural performance is compared to that of a non-wrapped RC column, which measures two meters in height with a 300 mm square cross-section and is reinforced with four main bars, 20 mm in diameter, and seven stirrups per meter, 6 mm in diameter. The load-displacement relationships obtained for the investigated columns are presented in Fig. 15, illustrating the effect of various fiber wrapping on the columns’ stiffness and strength.

**Fig. 16:**
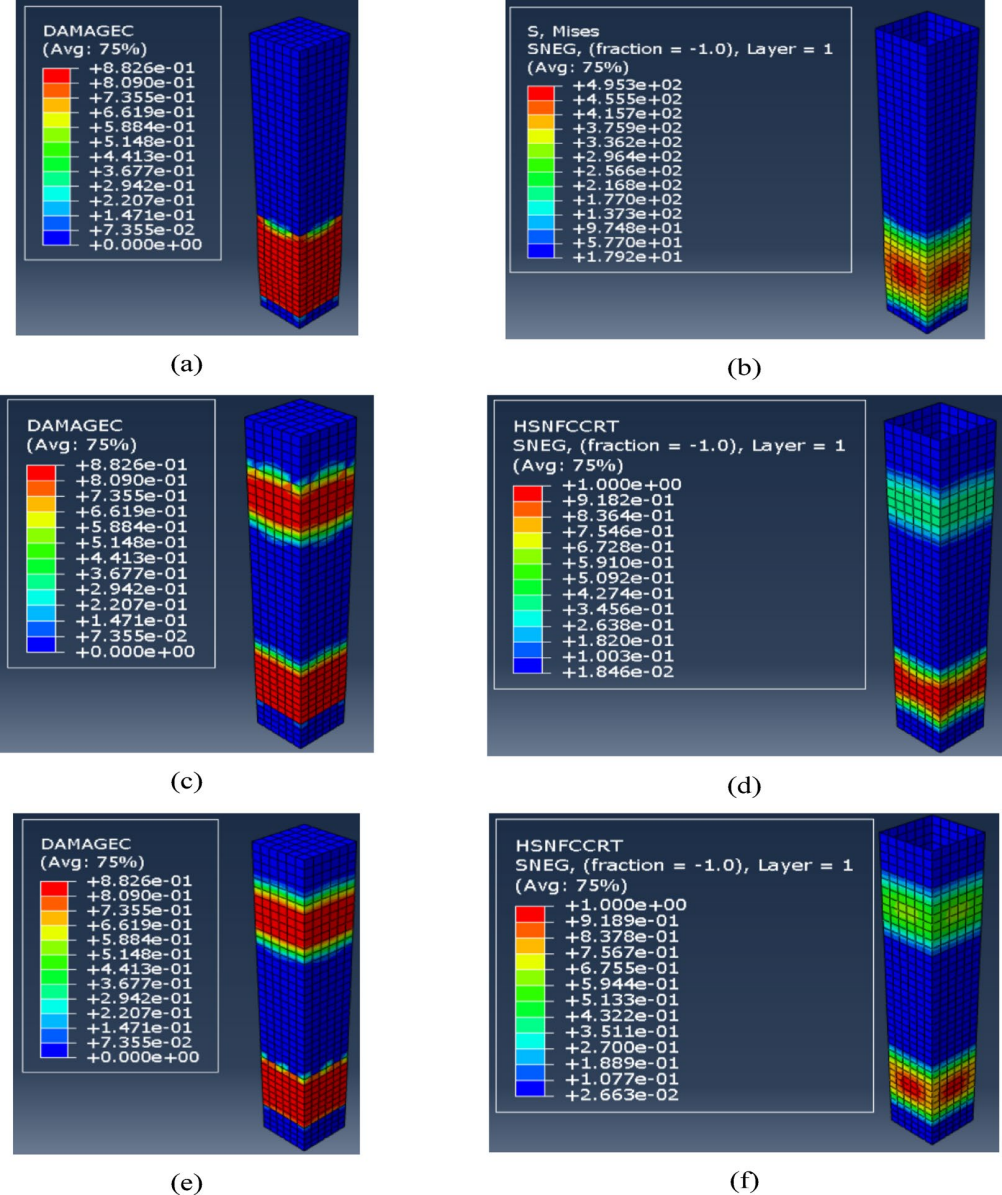
(**a**) Concrete compression damage for column with KFRP, (**b**) Stress in KFRP, (**c**) Concrete compression damage for column with GFRP, (**d**) Compression damage of GFRP (e) Concrete compression damage for column with CFRP, (**f**) Compression damage of CFRP.

Figure 15 illustrates that wrapping RC columns with various types of fiber laminates has negligible impact on the initial stiffness, which is determined by the modulus of elasticity of the concrete material. Furthermore, the increase in the ultimate strength of the wrapped columns is minimal, as the control column exhibited an ultimate load of 2803 KN, the application of KFRP, GFRP, and CFRP increased the ultimate loads to 2852 KN, 2881 KN, and 2976, respectively. The failure modes of the columns could be illustrated by the compressive damage of the concrete material and the fiber wrapping. Figure 16 illustrates the failure of the wrapped columns utilizing the three types of fibers under investigation.

## Results and discussions

**Fig. 17:**
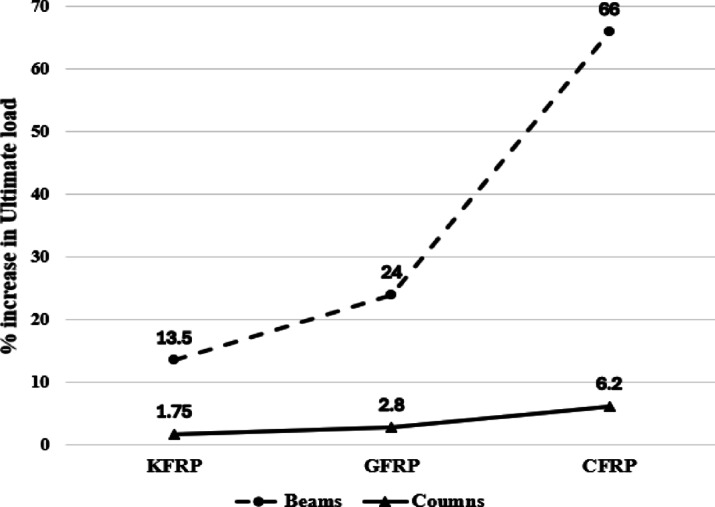
Effect of fiber wrapping on a percentage increase in ultimate loads for beams & columns.

The analysis of the results presented in the preceding sections demonstrates the enhanced performance of KFRP, GFRP, and CFRP reinforced beams in comparison to the control beam. Wrapping the RC beam with fiber laminates led to an enhancement in the ultimate load by 13.5%, 24%, and 66% for KFRP, GFRP, and CFRP, respectively. Furthermore, wrapping the RC beam with fiber laminates led to a decrease in the deflections of the beams under comparable loads to the control beam. Furthermore, the ductility of the beams, as shown by the toughness ratio, improved by 19.47%, 42.88%, and 72.09% for KFRP, GFRP, and CFRP, respectively. Conversely, the effect of FRP wrapping on RC columns yields lesser enhancements in ultimate load capacity than on beams, with the wrapping of RC columns leading to increases in ultimate load of 1.75%, 2.8%, and 6.2% for KFRP, GFRP, and CFRP, respectively. Figure 17 illustrates a comparison of the % increase in ultimate loads for both beams and columns resulting from fiber wrapping.

The disparity in behavior between beams and columns when subjected to fiber wrapping can be explained by the fundamental behavior of these structural elements. The improved performance of RC beams is due to the fibers’ capability to confine the concrete and withstand tensile stresses, which are critical in bending-dominated elements such as beams. The FRP wraps significantly improve the tensile strength of the beams and postpone the initiation of cracking and failure. The enhanced tensile strength directly correlates to the substantial improvements in the ultimate load capacity and ductility of the beams. Moreover, the decrease in deflection under comparable loads emphasizes the stiffness improvement provided by the wraps. Short columns predominantly undergo axial compression, and the enhancement in compressive strength achieved through FRP wrapping is typically less pronounced than the improvements in tensile strength observed in beams. The modest increases in ultimate load for wrapped columns are partly attributed to the substantial inherent compressive capacity of concrete. Moreover, the limited enhancement observed in the wrapped RC columns can be explained by the effectiveness of FRP confinement, which is strongly dependent on the cross-sectional shape of the column. FRP confinement is most efficient in circular columns, where continuous wrapping generates uniform lateral confining pressure and fully mobilizes the hoop tensile strength of the fibers. In contrast, square and rectangular columns, such as those investigated in this study, exhibit non-uniform confinement, with stress concentrations developing at the corners and significantly reduced confinement effectiveness along the flat faces, leading to partial utilization of the FRP capacity, Lam & Teng^53^. Consequently, FRP wrapping in non-circular columns primarily delays brittle failure rather than significantly increasing axial load capacity, which is consistent with the numerical findings of the present study.

The results clearly demonstrate the enhanced performance of CFRP reinforced beams in comparison to GFRP and KFRP, establishing it as a viable option for strengthening structural elements. KFRP exhibits less significant performance enhancements relative to GFRP and CFRP; yet it still offers substantial advantages. KFRP, as a sustainable material, corresponds with the worldwide movement towards sustainability and diminishing dependence on non-renewable resources. Its sustainable characteristics render it especially appealing for green building certifications. Furthermore, although KFRP demonstrates lesser ultimate load and ductility enhancements (13.5% and 19.47%, respectively), its improvements remain significant for numerous practical applications. It can be utilized in structures requiring moderate strengthening or in less critical areas, while GFRP or CFRP can be allocated to high-stress zones, thereby optimizing material utilization and minimizing environmental impact.

## Conclusion

A comprehensive FE model was generated utilizing ABAQUS software to examine the behavior of a simply supported RC beam. A FE modelling protocol is presented, outlining numerous modelling considerations, including solution approaches, element types, FE meshing, boundary conditions, material modelling, and interactions among various components within the model. The FE model was validated by comparing its results with prior numerical and experimental studies. The validated model was subsequently utilized to perform comparative analysis to assess the impact of fiber wraps on the structural performance of RC beams and columns, specifically evaluating the degree to which fiber wrapping enhances the behavior of these elements and determining which element exhibits greater enhancement. The study examines the impact of several fiber materials, including CFRP and GFRP, to identify the most effective type for structural strengthening. The research evaluates the viability of sustainable, green fibers by utilizing kenaf fibers and comparing their effectiveness with conventional fiber materials. Eight models were developed for beams and columns to examine their structural behavior with the above-mentioned fiber laminates.

The subsequent points could summarize the main findings:

- Wrapping RC beams with FRP significantly enhances their load-carrying capability, mostly due to the substantial improvement in tensile strength resulting from fiber contribution.
- Wrapping RC beams with fiber laminates resulted in an increase in the ultimate load by 13.5%, 24% and 66% for KFRP, GFRP and CFRP, respectively.
- Wrapping the RC beam with fiber laminates led to a decrease in the deflections of the beams under comparable loads to the control beam.
- The ductility of the beams increased by 19.47%, 42.88%, and 72.09% for KFRP, GFRP and CFRP, respectively.
- Wrapping RC columns with FRP typically results in smaller improvements in ultimate load capacity compared to beams, where wrapping the RC column resulted in an increase in the ultimate load by 1.75%, 2.8% and 6.2% for KFRP, GFRP and CFRP, respectively.
- The overall performance of CFRP was superior within the investigated configurations, indicating its effectiveness as a strengthening solution for RC beams and columns under the conditions studied.
- KFRP may not achieve the performance levels of GFRP or CFRP; nonetheless, its sustainability and cost-effectiveness render it a viable choice for applications prioritizing environmental concerns and economic efficiency. Throughout a structure’s lifecycle, the environmental benefits of KFRP, such as diminished consumption of energy and reduced waste, may surpass the small improvements in mechanical performance.

### Limitations and future work

The findings of this study should be interpreted within the scope of its numerical framework, as the investigation was based on FE simulations calibrated against existing studies without new experimental validation. The analyses were limited to specific beam and column geometries, loading conditions, and boundary configurations. In addition, constant laminate thickness was adopted across all models to isolate the influence of fiber material type. Future research should incorporate experimental validation and investigate stiffness-, weight-, or cost-equivalent FRP configurations, as well as the influence of different laminate thicknesses, confinement layouts, and cross-sectional geometries. Extending the analysis to include comprehensive life-cycle sustainability assessments would provide a more complete evaluation of natural FRP systems for structural strengthening applications.

## Data Availability

This data is available on request from the corresponding author.
